# Transcriptomic analysis of bovine endometrial epithelial cells in response to interferon tau and hormone stimulation

**DOI:** 10.3389/fvets.2024.1344259

**Published:** 2024-02-02

**Authors:** Jie Yu, Chenhui Liu, Hongbo Chen, Min Xiang, Xiuzhong Hu, Zhuxia Zhong, Qing Liu, Dingfa Wang, Lei Cheng

**Affiliations:** ^1^Institute of Animal Science and Veterinary Medicine, Wuhan Academy of Agricultural Sciences, Wuhan, China; ^2^Laboratory of Genetic Breeding, Reproduction and Precision Livestock Farming and Hubei Provincial Center of Technology Innovation for Domestic Animal Breeding, School of Animal Science and Nutritional Engineering, Wuhan Polytechnic University, Wuhan, China

**Keywords:** bovine, early pregnancy, endometrial epithelial cells, interferon tau, transcriptomic profiling

## Abstract

The embryonic loss during early stage of gestation is one of the major causes of infertility for domestic ruminants, causing huge economic losses to pasture. Maternal recognition of pregnancy and implantation are the crucial process for determining the successful establishment and development of pregnancy in cattle. The research on molecular mechanisms of pregnancy recognition will facilitate illustrating the complex process of pregnancy establishment and help to improve pregnancy outcomes. In this study, we performed transcriptomic analysis of primary bovine endometrial epithelial cells (BEND) with or without IFNT and hormones intervention through RNA sequencing. We eventually identified 608 differentially expressed genes (DEGs) including 409 up-regulated genes and 199 down-regulated genes in IFNT and hormones-treated group compared with control group. Gene Ontology (GO) enrichment analysis demonstrated that the majority of DEGs were implicated in immune system process, response to external stimulus, response to cytokine, regulation of response to stress. Results from KEGG analysis showed a significant enrichment of NOD-like receptor signaling pathway, antigen processing and presentation, necroptosis, oxidative phosphorylation, RIG-I-like receptor signaling pathway. Additionally, a set of promising candidate genes, including (*USP18*, *STAT1*, *PSMB8*, *IFIH1*, *MX2*, *IFI44*, *DHX58*, *CASP8*, *DRAM1*, *CXCR4*), were characterized by constructing an integrated interaction network. Specifically, the mRNA expression of *HOXA11*, *PTGS1* and *PTGS2* were remarkably suppressed by silencing *DRAM1* under IFNT and hormone administration, thus speculating that *DRAM1* might play a crucial role in early pregnancy by regulating endometrial function. The results of this study depicted a relatively comprehensive transcriptional profiles of BEND in response to IFNT and hormones, which contributes to a better understanding of gene interaction network and underlying regulatory mechanisms in endometrium of ruminants during early pregnancy.

## 1 Introduction

Reproductive performance is a key determinant affecting the economic benefits of pasture. Pregnancy failure will retard the reproductive cycles, increase feeding costs and cause enormous economic losses in the livestock sector. In domestic ruminants, the establishment of pregnancy involves multiple physiological and biochemical processes, including pregnancy recognition, embryo implantation and placentation. It is well known that the fertilization rate in ruminants is considerably higher than pregnancy rate due to the vast majority of pregnancy loss occurring during the peri-implantation period, which encompasses the maternal recognition of pregnancy process ranging from days 16 to 25, contributing to approximately 30–35% embryo loss in cattle (1–4). Maternal pregnancy recognition (MRP) is a physiological process whereby the embryo signals its presence to maternal system, thus preventing the regression of corpus luteum (CL), prolonging the lifespan of CL and secreting progesterone (P_4_) continuously (5, 6). Therefore, the moderate communication between conceptus and maternal uterus is crucial for the establishment and maintenance of pregnancy.

With regard to the establishment of pregnancy in ruminants, endometrial epithelium requires the coordinated orchestration of conceptus-derived interferon-tau (IFNT), and maternally derived estradiol (E_2_) and P_4_ (7–9). IFNT is the primary signal of pregnancy recognition, exclusively secreted by mononuclear cells of conceptus trophectoderm during the early gestation period in ruminant species (10). Accompanied by the progression of pregnancy, IFNT is expressed from days 10 to days 21–25 with maximal peaks on days 17–20 in cattle, which acts on the endometrium through a paracrine manner and causes changes of prostaglandin secretion patterns during pregnancy recognition (11). The expression of estrogen receptor (ESR1) and oxytocin receptor (OXTR) are suppressed by IFNT in endometrial epithelial cells, thereby preventing the luteolytic pulses of prostaglandin F_2α_ (PGF_2α_) from uterus (12). Moreover, P_4_ can inhibit the expression of ESR1 by binding to progesterone receptor (PR), subsequently downregulating OXTR in endometrial luminal and superficial glandular epithelial cells (13). The combinatorial and sequential action of P_4_ and IFNT ultimately restrains the expression of ESR1 and OXTR in endometrium, which inhibits the release of PGF_2α_ and prevents luteolysis. IFNT can also significantly increase the secretion of PGE_2_ in endometrial stromal cells to prohibit luteolysis and maintain pregnancy (14). In addition, various available evidences indicated that IFNT regulate the expression of numerous P_4_-induced genes implicated in embryonic development, implantation, uterine receptivity and immune tolerance (15–18). The expression of several classical interferon stimulated genes (ISGs) in endometrium such as *ISG15*, *RSAD2* and *OAS1* can be upregulated by IFNT through the JAK–STAT-IRF signaling pathway, that play an essential role in modulating conceptus elongation, implantation and immunity during pregnancy (19). IFNT also signal via MAPK, PI3K signaling pathway independent of STAT1 to regulate the expression of multiple non-canonical ISGs including *WNT7A*, *CST3*, *HIF2A* associated with endometrium-secreted cytokines that promote the proliferation and migration of trophectoderm cells (20). During the peri-implantation period of pregnancy, the nutrients in uterine histotroph are required for embryonic survival, development and adhesion. The expression of glucose transporters *SLC2A1*, *SLC5A1* and *SLC5A11*, as well as amino acid transporters *SLC7A1* and *SLC7A2* are remarkably enhanced in endometrial luminal and superficial glandular epithelium of ewes from days 10 to 20 of pregnancy under the stimulation of P_4_ and IFNT, which functions prominently for conceptus growth and elongation (21, 22). A recent study revealed that the expression of *CASP7*, *CASP8*, *CASP4* and *NLRP3* in uterus from 18-day gestation cattle are dramatically higher than that of non-pregnant uterus, and IFNT also stimulates the expression of *CASP8* and *CASP11* in endometrial epithelial cells (23), the apoptosis and pyroptosis-related genes are speculated to involve in IFNT-induced pregnancy recognition and embryo implantation. While plenty of genes have been identified during peri-implantation period in ruminants, the gene-interaction network and underlying molecular mechanisms regulating MRP remain intricate, which need to be further elucidated. Herein, RNA-seq was applied to dissect the changes of gene expression profile in bovine endometrial epithelial cells (BEND) under the combinatory treatment of IFNT, P_4_ and E_2_, the regulatory network during MRP was uncovered by metabolic pathway analysis, that provides a reference for clarifying the molecular mechanisms of MRP in cattle.

## 2 Materials and methods

### 2.1 Bovine endometrial epithelial cell isolation and primary culture

Fresh and healthy uteri without endometritis from Holstein cows were acquired from a surrounding abattoir (Wuhan, China). The health and estimated estrous cycle of bovine were objectively confirmed by individual records of well-being and examination of veterinary surgeon from Wuhan Academy of Agricultural Sciences. This experimental processes were approved by the Institutional Animal Use Committee of Institute of Animal Science and Veterinary Medicine, Wuhan Academy of Agricultural Sciences.

Primary bovine endometrial epithelial cells were isolated and cultured in accordance with protocols previously described with some minor changes (24, 25). The uteri were eviscerated within 30 min after exsanguination, and immediately rinsed 3 times with ice-cold PBS to remove residual blood and dirt on the surface. After be immersed in sterile PBS, the uteri were kept on ice and transported to the laboratory within 1 h. The endometrium tissue was dissected into strips, which were placed in serum-free DMEM/F12 (Gibco, United States) supplemented with 50 U/L penicillin, 50 μg/mL streptomycin (Gibco, United States), 50 μg/mL gentamycin (Sangon Biotech, Shanghai, China) under sterile conditions. Then, the strips were cut into about 1mm^3^ pieces and washed 3 times with sterile PBS. Tissue pieces were digested with PBS containing 0.1% collagenase I (Sigma, United Kingdom) and incubated in a constant-temperature shaker at 80 r/min for 1 h at 37°C. The suspension was filtered with a 40 μm mesh, the filtrate was centrifuged to remove the supernatant at 1000 r/min for 10 min at room temperature, and resuspended in DMEM/F12 supplemented with 10% FBS (Gibco, United States). After washing five times, the cell pellets were resuspended with complete culture medium composed of 90% DMEM/F12, 10% FBS, 50 U/L penicillin and 50 μg/mL streptomycin, and dispersed into single cell suspension. Cells were seeded into a 10 cm culture dish which was kept in a water-jacked incubator at 37°C with 5% CO_2_. The medium was changed every 48 h. Subculturing was conducted when the cells reached approximately 80–90% confluence. Cell morphology was recorded and photographed under an inverted microscope.

The endometrial epithelial cells were purified by differential attachment method following three to five passages. Then, the purity of epithelial cell culture was confirmed by immunofluorescence detection of cytokeratin 18. Briefly, the primary endometrial epithelial cells were plated on glass coverslips in six-well culture dishes. The cells were fixed in 4% paraformaldehyde for 15 min after reaching 50–60% confluence, and permeabilized with 0.5% Triton X-100 (prepared in PBS) for 20 min at room temperature. Non-specific binding was blocked by pre-treating the cells with 3% BSA (Sigma, United States) for 30 min at room temperature. Subsequently, the cells were incubated with primary antibody CK-18 (Servicebio, Wuhan, China) at 4°C overnight, followed by incubation with the secondary fluorescently-labeled Cy3 antibody (Servicebio, Wuhan, China) for 1 h at room temperature, after which the cells were washed three times in PBS. The nuclei were counterstained with DAPI (Thermo, United States). Fluorescent images were obtained using a laser scanning confocal microscope (NIKON Eclipse Ti, Tokyo, Japan).

### 2.2 Cell treatment

**Figure 1:**
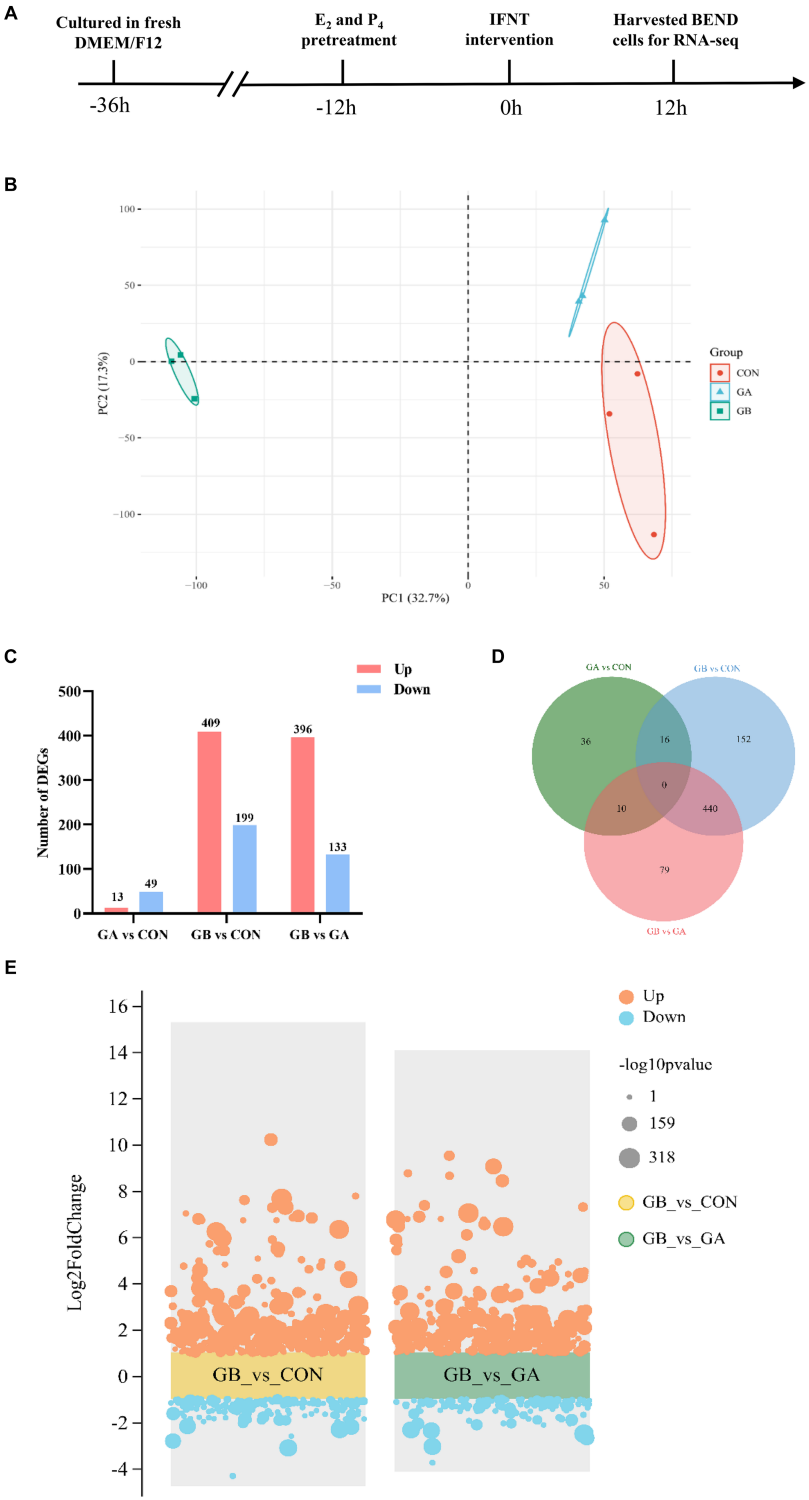
Identification of differentially expressed genes. **(A)** The workflow of sample preparation for sequencing; **(B)** PCA analysis of the expressed transcripts. The *X*-axis is the first principal component, and the *Y*-axis is the second principal component. Different shapes and colors represent different groups; **(C)** Statistical analysis of DEGs in GA vs. CON, GB vs. CON, GB vs. GA groups; **(D)** Venn diagram of the number of common DEGs in GA vs. CON, GB vs. CON, GB vs. GA groups; **(E)** Volcano plot of DEGs identified in GB vs. CON, GB vs. GA groups. The orange and blue dots represent up- and down-regulated genes, respectively. The dots size indicates the corresponding -log10*p* value, a larger dot represents a smaller *P* value.

As shown in Figure 1A, the BEND at logarithmic growth stage were inoculated in 6 cm dishes and cultured with DMEM/F12 containing 10% FBS and 1% penicillin–streptomycin in humidified atmosphere with 5% CO_2_ at 37°C. Once reaching 50–60% confluence, the cells were divided into three groups of three replicates and rinsed with PBS. For the groups specified for E_2_, P_4_ and IFNT treatment, the medium were replaced by fresh complete culture medium supplemented with E_2_ (10^−9^ M, Sigma) and P_4_ (10^−7^ M, Sigma, United States), and continued culturing for 12 h. Subsequently, the cells were treated with IFNT (100 ng/mL, Cell sicences, United States) for an additional 12 h.

### 2.3 RNA extraction and quality assessment

Total RNA extraction from BEND were performed with TRIzol reagent (Invitrogen, United States), and genomic DNA was removed using DNase I (Invitrogen, USA) according to the manufacturer’s instructions (HiPure Universal RNA Mini Kit, Magen). RNA degradation and contamination were monitored on 1% agarose gels. The quantity and purity of RNA was measured using Qubit 3.0 (Thermo Fisher Scientific, MA, United States) and Nanodrop One (Thermo Fisher Scientific, MA, USA). RNA integrity was accurately assessed using the Agilent 2,100 system (Agilent Technologies, Waldbron, Germany).

### 2.4 Library preparation and sequencing

The mRNAseq library was generated using the ALFA-SEQ Directional RNaLib Prep Kit following the manufacturer’s recommendations. In brief, mRNA was purified from total RNA using poly-T oligo-attached magnetic beads. Fragmentation was performed using RT Buffer interruption within the ALFA-SEQ Directional RNaLib Prep Kit. First-strand cDNA was synthesized using random hexamer primers and M-MuLV reverse transcriptase (RNase H), followed by second-strand cDNA synthesis using DNA polymerase I and RNase H. The remaining overhangs were converted into blunt ends by exonuclease/polymerase activity. After adenylation of the 3′ end of the DNA fragments, X-Fold diluted VAHTS Adapter-S with a hairpin loop structure were ligated to prepare for hybridization. To preferentially select cDNA fragments of 150–200 bp in length, these library fragments were selected by AMPure XP beads (GE Sera-Mag Magnetic Speed-beads carboxyl-modified). Then, PCR was performed with 2× PfuMax HiFi PCR ProMix (EnzyValley), primers with index VAHTS Multiplex Oligos Set 4 for Illumina. Finally, AMPure XP beads (GE Sera-Mag Magnetic Speed-beads carboxyl-modified) was used to purify the PCR products, and the library insert sizes were evaluated on the Qsep400 high-throughput nucleic acid protein analysis system (Houze Biotechnology Co., Ltd., Hangzhou, China). The clustering of the index-coded samples was performed on a cBot Cluster Generation System. After cluster generation, the library preparations were sequenced on an Illumina Hiseq 2,500 platform and 150 bp paired-end reads were generated. The sequencing data generated in this study were deposited in the NCBI GEO database (accession number GSE253481).

### 2.5 Data quality control and reference genome alignment

Use fastp (v0.21.0) (https://github.com/OpenGene/fastp) to process the raw data in fastq format to obtain clean data (clean reads). Clean reads were mapped to the NCBI Rfam database, and rRNA sequences were removed by Bowtie2 (v2.33) (https://github.com/BenLangmead/bowtie2). The reference genome (ARS-UCD1.2) and annotation files were downloaded directly from the genome website. The sequences after ribosome removal were aligned to the reference genome by Hisat2 (v2.2.1) software (https://github.com/ infphilo/ hisat2).

### 2.6 Differential expression analysis

RSEM (v1.3.3) (https://github.com/deweylab/RSEM) was applied to obtain the read counts for each gene. Fragments per kilobase of exon per million mapped reads (FPKM) of genes in each sample were calculated to estimate gene expression levels. Differentially expressed genes between two groups (three biological replicates per group) were screened with DESeq2 package (v1.34.0) (http://www.bioconductor.org/packages/release/bioc/html/DESeq2.html). The resulting *p*-values were adjusted using the Benjamini-Hochberg method for controlling the false discover rate (FDR). |log2 (Fold Change)| > 1 and FDR < 0.05 were set as thresholds for significantly differential expressed genes (DEGs). These genes were used for subsequent bioinformatic analysis.

### 2.7 GO and KEGG enrichment analyses of differentially expressed genes

The function annotation of DEGs, including cellular component (CC), molecular function (MF) and biological process (BP), were analyzed using Gene Ontology (GO) database (http://www.geneontology.org). Kyoto Encyclopedia of Genes and Genomes (KEGG) database (http://www.genome.jp/kegg/) was engaged to systematically analyze the biochemical metabolic and signal transduction pathways in which the DEGs are involved. GO and KEGG enrichment analysis of DEGs were performed using the clusterProfiler (v4.2.2) (http://www.Bioconductor.org/packages/release/bioc/html/clusterProfiler.html) for function and signaling pathway annotation. GO terms and KEGG pathways with FDR < 0.05 were considered significantly enriched for DEGs.

### 2.8 PPI network analysis

A Protein–protein interaction (PPI) network was constructed for DEGs, based on the STRING database (http://string-db.org). The constructed networks were based on known interactions in the selected reference species (cow) with a combined interaction score above 0.4 as selection threshold. Cytoscape software (version 3.10.1) was applied to visualize the PPI networks.

### 2.9 qRT-PCR

The expression profiles of selected DEGs were validated to confirm the repeatability and reproducibility of gene expression data obtained by RNA sequencing, which were accomplished by SYBR Green-based qRT-PCR using specific primers designed by an online tool (https://primer3.ut.ee/) and a subset of samples from those utilized for RNA sequencing. 1 μg of RNA was reverse transcribed into cDNA, using a reverse transcription kit (with gDNA wiper, Vazyme, China) following the manufacturer’s instructions. The relative expression level of genes were determined by qRT-PCR using the AceQ qPCR SYBR Green Master Mix (Vazyme, China) with the following program: pre-degeneration at 95°C for 5 min, 40 cycles of degeneration at 95°C for 10s and annealing at 60°C for 30s. Data acquisition and analysis were performed on a CFX96 Connect real-time PCR detection system using CFX manager software (Bio-Rad, United States). The sequences of primers synthesized by Tsingke Biotechnology Co., Ltd. (China) are listed in Supplementary Table S1. Relative mRNA expression levels of target genes were normalized against the relative quantity of *ACTB* mRNA, which was based on threshold cycle (Ct) values using a comparative 2^-△△^Ct method. qRT-PCR was carried out in three biological replicates.

### 2.10 RNA interference

RNA interference of *DRAM1* was executed using small interfering RNA (siRNA). BEND was transiently transfected with control siRNA or siRNA-DRAM1 by Lipofectamine 3,000 (Invitrogen, United States) according to the manufacturer’s manual. The sequence of siRNA- DRAM1 was sub5’-UCCUGCAAUCCGUCAUCUCUUdTdT-3′. qPCR was employed to detect the knockdown efficiency of *DRAM1* in BEND.

### 2.11 Statistical analysis

Experimental data are presented as mean ± standard deviation (SD). All data were representative for one of three independently repeated experiments. The statistical analyses were carried out by GraphPad Prism software 8.0 (GraphPad Prism Software Inc., CA, United States). Unpaired two-tailed t test (Student’s *t*-test) was devoted to make comparisons between two independent groups. For comparison of statistical differences among multiple groups (>2), one-way analysis of variance (one-way ANOVA) with multiple comparisons tested by Tukey’s *post hoc* test was performed. *p* < 0.05 was considered statistically significant.

## 3 Results

### 3.1 Identification of differentially expressed genes

The identification of primary BEND was performed by immunofluorescence, which showed that the epithelial-specific marker cytokeratin is positive in primary BEND (Supplementary Figure S1). The flow schema of sample preparation for sequencing is shown in Figure 1A. Triplicate mRNAseq libraries from three groups of BEND, which includes cells of control group (CON), cells treated with E_2_ and P_4_ (GA), cells treated with E_2_, P_4_ and IFNT (GB), were generated and sequenced to yield 65.15Gb of data. After filtering the sequencing adapter sequence and low-quality reads, a total of 62,289,909, 67,196,670, 63,332,621 clean reads with >93.67% of Q30 and exceeding 51.74% of the GC content were acquired from CON, GA and GB group, respectively, that were used for the succedent analyses. The mean value of clean reads ratio was 88.66, 88.25 and 89.55% for the CON, GA and GB group, respectively (Supplementary Table S2). Approximately 98.18–98.38% of clean reads were mapped to the reference genome after removal of reads aligned with rRNA (Supplementary Table S3). Unmapped reads (1.62–1.82%) were excluded from further analyses. Accordingly, 17,162 mRNA transcripts were detected in the 9 samples. Principal component analysis (PCA) which clusters similar samples together and accounts for the origin of variance in data was performed with two elements: PC1 (32.7%) and PC2 (17.3%), indicating high similarity between samples in the group (Figure 1B). All these results demonstrated the reliability of RNA sequencing data, that could be used in further analyses.

The differential gene expression profile between groups was determined with DESeq. A total of 608 DEGs were identified in GB group compared to the CON group, among which 409 genes were upregulated and 199 genes were downregulated; 529 DEGs composed of 396 upregulated genes and 133 downregulated genes were identified in GB group compared to the GA group; 62 DEGs containing 13 upregulated genes and 49 downregulated genes were detected in GA group compared to the CON group (Figure 1C). In addition, a venn diagram of DEGs in three comparison groups was plotted, which showed that 440 genes are co-differentially expressed in both comparison groups (GB vs. CON, GB vs. GA) (Figure 1D). The volcano plot was constructed to visualize the distribution and fold change of these DEGs identified in GB vs. CON and GB vs. GA (Figure 1E) groups.

### 3.2 Cluster analysis of differentially expressed genes

**Figure 2:**
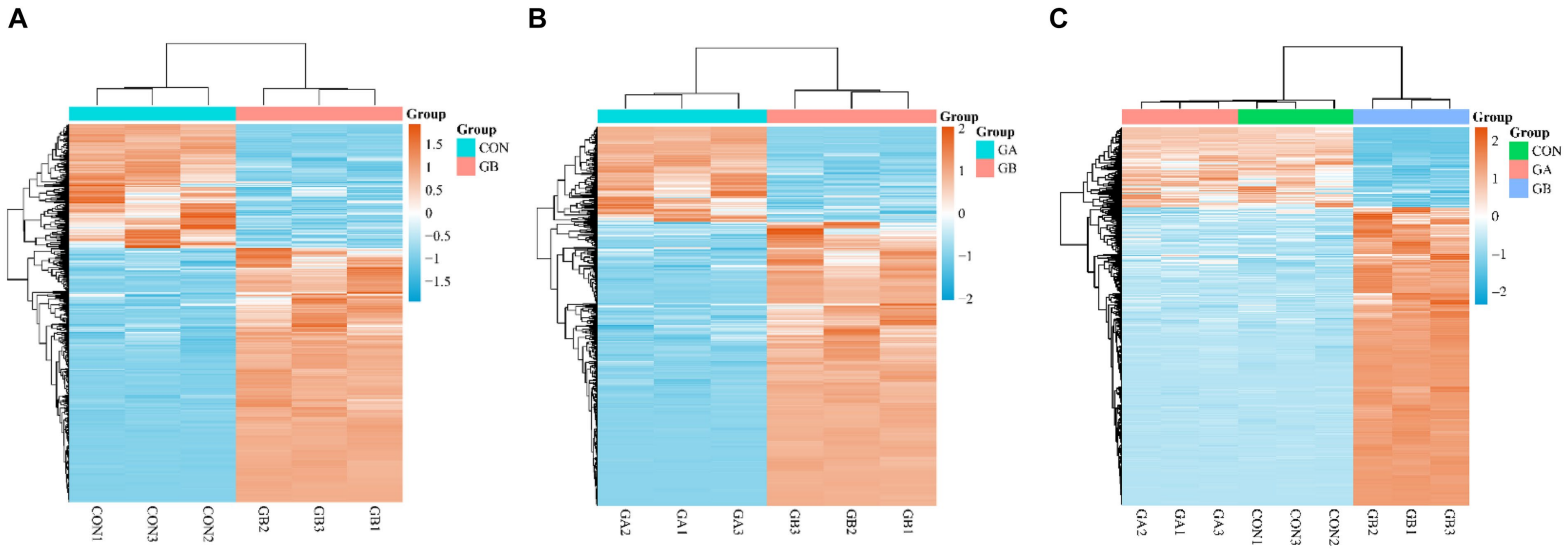
Cluster analysis of DEGs. Heatmap of DEGs in the pair of GB vs. CON **(A)**, GB vs. GA **(B)**, common DEGs in GB vs. CON and GB vs. GA **(C)**. The expression level was calculated by log2 (FPKM) presented as gradient color barcode at the right top. The orange and blue color indicate up- and down-regulated genes, respectively.

The hierarchical cluster analysis of DEGs was applicated to identify the expression level of genes with high correlation among samples. Heatmaps depicting the DEGs of GB vs. CON, GB vs. GA groups are shown in Figures 2A,B, respectively. Meanwhile, we plotted a heatmap of 440 DEGs that were commonly present in both comparison groups, which revealed that most of the DEGs exhibit similar differential expression trends and patterns among samples (Figure 2C).

### 3.3 GO and KEGG enrichment analyses

**Figure 3:**
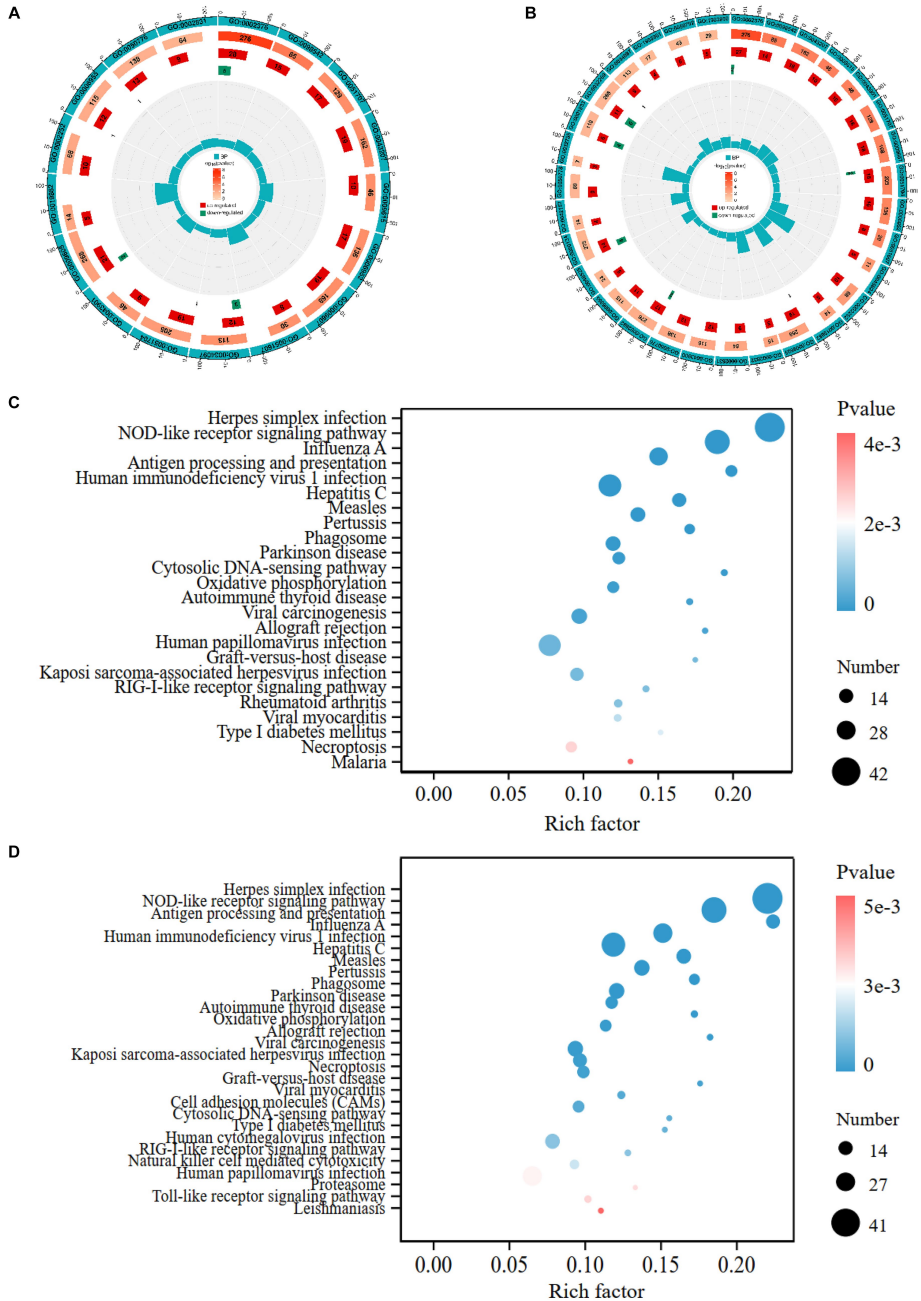
GO and KEGG enrichment analyses of DEGs. The circle diagrams of GO enrichment analysis of DEGs in the pair of GB vs. CON **(A)**, GB vs. GA **(B)**. The first circle shows significantly enriched GO term from biological process ontology; the second circle indicates the number and value of p of the GO term in each classification gene background; the third and fourth circle indicate the number of up- and down-regulated genes in each category of GO term, respectively; the fifth circle indicates enrichment factor value of each GO term. The bubble plots display the significantly enriched KEGG pathways in the pair of GB vs. CON **(C)**, GB vs. GA **(D)**. The size of dot represents the number of DEGs annotated in each pathway term, the color of dot denotes *p*-value.

We performed gene ontology (GO) annotation of DEGs to characterize the functional roles of these genes in biological pathways. For GO enrichment analysis, GO terms with adjusted *p*-value <0.05 were designated as significantly enriched. A large number of DEGs were enriched in the GO biological process category. 17 GO terms from Process Ontology were significantly enriched in GB vs. CON group, such as immune system process, response to external biotic stimulus, defense response, response to cytokine, antigen processing and presentation (Supplementary Table S5). 43 GO terms from Process Ontology were significantly enriched in GB vs. GA group, including immune system process, multi-organism process, regulation of immune system process, regulation of response to stress, negative regulation of cellular protein metabolic process (Supplementary Table S6). The GO terms with most significant enrichment in GB vs. CON (Figure 3A), GB vs. GA (Figure 3B) are displayed as circle diagrams.

KEGG enrichment analysis of DEGs was conducted to calculate the abundant metabolic pathways and signal transduction pathways. KEGG pathways were considered significantly enriched with adjusted *p*-value <0.05. Overall, 348 DEGs were assigned to 24 significantly enriched KEGG pathways in GB vs. CON group, which contains herpes simplex infection, NOD-like receptor signaling pathway, influenza A, antigen processing and presentation, measles, parkinson disease, cytosolic DNA-sensing pathway, oxidative phosphorylation, RIG-I-like receptor signaling pathway, necroptosis (Supplementary Table S7). Moreover, 305 DEGs were allotted to 28 significantly enriched KEGG pathways in GB vs. GA group, such as herpes simplex infection, NOD-like receptor signaling pathway, human immunodeficiency virus 1 infection, human papillomavirus infection, influenza A, phagosome, cell adhesion molecules (CAMs), Toll-like receptor signaling pathway (Supplementary Table S8). Advanced bubble charts reveal the significantly enriched KEGG pathways of DEGs in GB vs. CON (Figure 3C), GB vs. GA (Figure 3D).

### 3.4 PPI network analysis of differentially expressed genes

**Figure 4:**
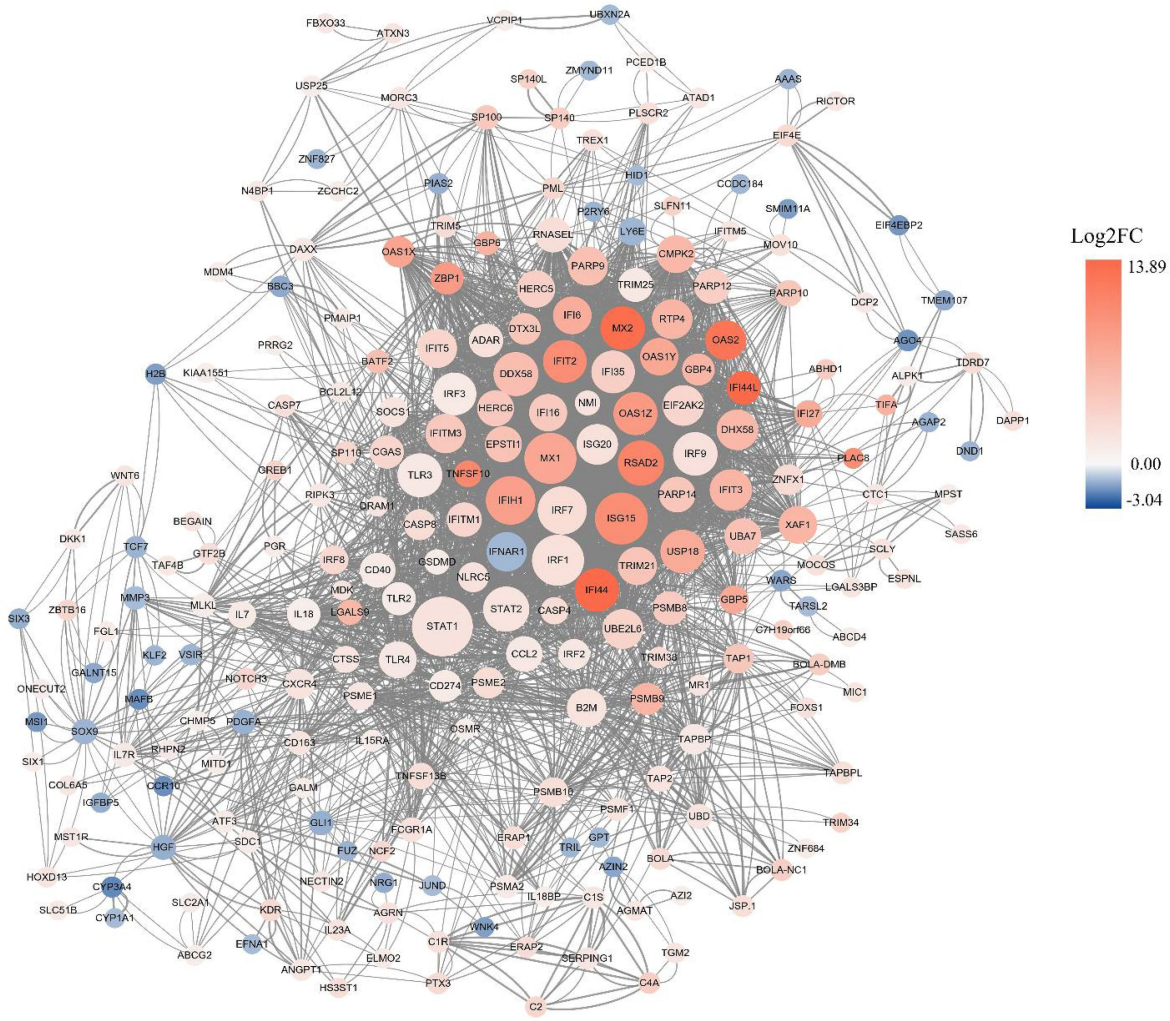
PPI network analysis of DEGs. The red and blue dots indicate up- and down-regulated genes, respectively. The intensity of color denotes fold-change of DEGs, and the size of dots represent node degree.

A PPI network composed of 260 nodes connected via 2018 edges was generated by Cytoscape software based on STRING database to illustrate the interactions between DEGs. The results showed that a few node genes interact closely with other genes, including *STAT1*, *IFIH1*, *MX2*, *IFI44*, *USP18*, *DHX58*, *PSMB8*, *CASP8*, *DRAM1*, *CXCR4* (Figure 4). Some of these highly connected node genes are rarely reported to correlate with early pregnancy, that need to be concerned and further investigated.

### 3.5 qRT-PCR verification

**Figure 5:**
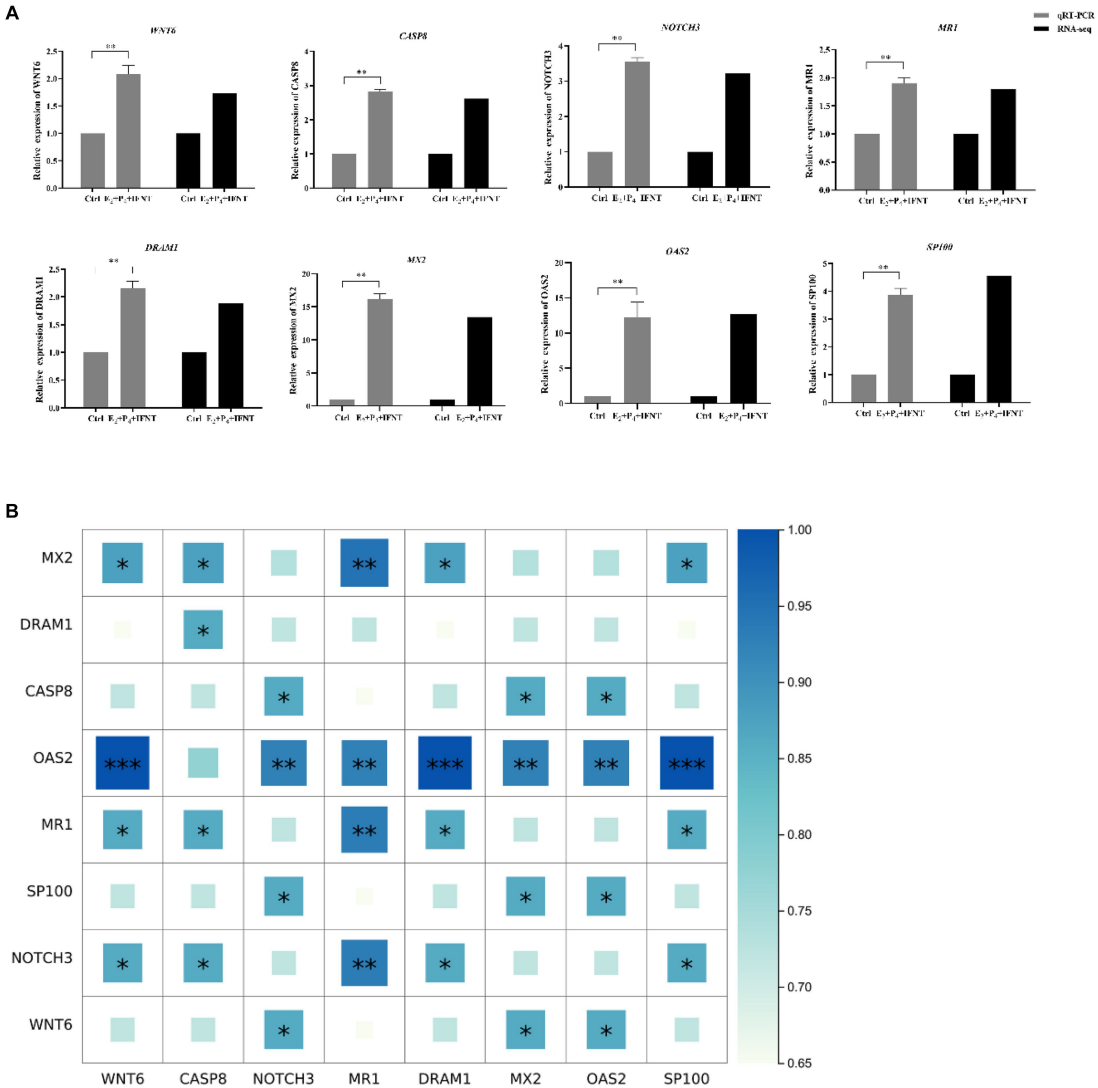
Expression pattern validation of selected DEGs by real-time PCR. **(A)** The relative expression level of target mRNA was calculated using the 2−^△△^Ct method and presented as the ratio of Log2 fold change between groups; **(B)** Spearman’s correlation analysis heatmap of RNA-seq and qRT-PCR data. “*” *p* < 0.05, “**” *p* < 0.01, “***” *p* < 0.001.

Considering the results of GO and KEGG enrichment analysis, 8 DEGs were randomly selected for exploring the relative expression pattern to validate the accuracy and reliability of RNA-Seq results by performing qRT-PCR. The expression trend of all these selected genes in the results of qRT-PCR were proved to be identical with RNA-seq data (Figure 5A). Meanwhile, the results of Spearman’s correlation analysis of all selected genes showed a strong positive correlation between RNA-seq data and qRT-PCR experiments (*r*^2^ > 0.65, *p* < 0.05), which confirmed the credibility of DEGs (Figure 5B).

### 3.6 Effects of *DRAM1* interference on expression of genes related to receptivity and prostaglandin synthesis in bovine endometrial epithelial cells

**Figure 6:**
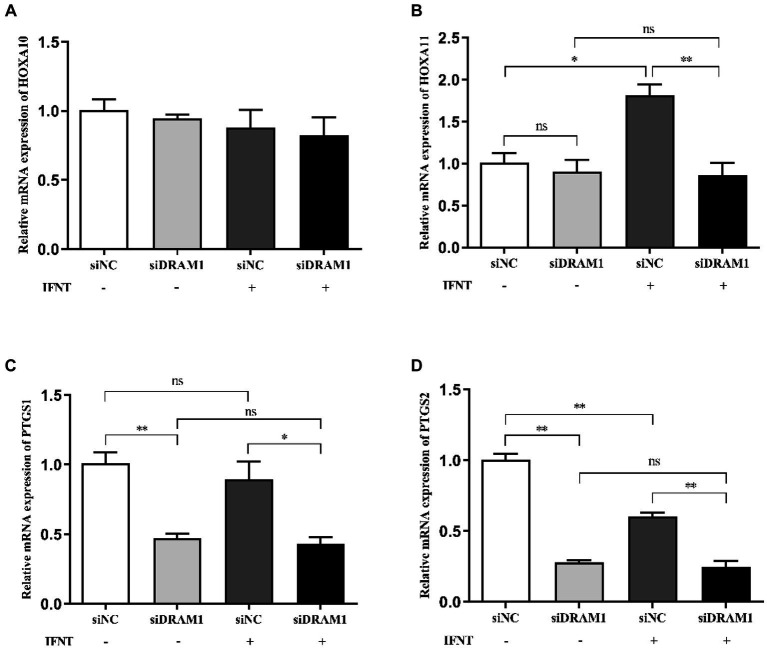
Effects of *DRAM1* interference on expression of genes related to receptivity and prostaglandin synthesis in BEND. BEND was transiently transfected with control siRNA or siRNA-DRAM1. Subsequently, the cells were incubated with E_2_ and P_4_ followed by IFNT stimulation for 12 h. Total RNA isolated form BEND were applied to Real-time PCR. Real-time PCR analysis of the mRNA expression of endometrial receptivity marker gene *HOXA10* **(A)** and *HOXA11* **(B)**. Real-time PCR analysis of the mRNA expression of rate-limiting enzymes of PGs synthesis *PTGS1* **(C)** and *PTGS2* **(D)**. “*” *p* < 0.05, “**” *p* < 0.01.

To investigate the functional role of *DRAM1* during early pregnancy in ruminants, we conducted RNA interference assay to downregulate the transcript expression of *DRAM1* in BEND. The results indicated that the mRNA expression of *DRAM1* is prominently reduced (Supplementary Figure S2). Additionally, the mRNA expression of *HOXA11*, *PTGS1* and *PTGS2* were dramatically downregulated by silencing *DRAM1* upon IFNT stimulation (Figures 6B–D), but no significant change was observed on mRNA expression of *HOXA10* by suppressing *DRAM1* under IFNT treatment (Figure 6A). Furthermore, even in the absence of external intervention with IFNT, the mRNA expression of *PTGS1* and *PTGS2* were also significantly inhibited in siDRAM1 group compared with siNC group (Figures 6C,D). Nevertheless, interfering with DRAM1 exerted no distinct influence on the mRNA expression of *HOXA10* and *HOXA11* in BEND without IFNT treatment (Figures 6A,B).

## 4 Discussion

Pregnancy, an important driver for economic efficiency of pasture, is a complicated physiological process that requires the precise coordination of multiple molecular mechanisms involving numerous genes. However, the gene-interaction network and underlying molecular mechanisms implicated in early pregnancy remain elusive. Although there have been a few reports on the regulation of endometrium by IFNT in domestic ruminants, most studies which were performed in an immortalized bovine endometrial cell line only analyzed the role of IFNT, irrespective of the impact of hormones during early pregnancy (26, 27). In this study, we isolated and cultured primary endometrial epithelial cells of bovine (BEND), and performed transcriptomic analysis of BEND in response to IFNT, P_4_ and E_2_, which depicted a relatively comprehensive DEGs profile based on the RNA-seq dataset. Comparing our results with previous studies, we noticed some similarities and inconsistencies. Zhao et al. (27) identified 839 upregulated DEGs and 148 downregulated DEGs in bovine endometrial epithelial cells under the stimulation of IFNT for 12 h compared with untreated groups. Chaney et al. (28) analyzed the bovine endometrial epithelial transcriptomic response to recombinant IFNT and identified 622 increased DEGs, 41 decreased DEGs, among which 247 of 622 upregulated DEGs and 9 of 41 downregulated DEGs appeared in our DEGs list, and were consistent with the gene expression trend in our study. Adhikari et al. (29) compared the transcriptomic profiles of endometrial tissue of pregnant heifers (day 15–17 of gestation) with cyclic (non-bred) heifers and identified 107 DEGs, 7 of top 40 DEGs in Adhikari’s study were matched to the DEGs list of GB vs. CON group in our study; simultaneously, 6 of 7 DEGs showed consistent expression trend with our data, 1 DEG exhibited completely opposite expression trend.

GO enrichment analysis indicated that the higher number of DEGs in our data were mainly implicated in immune system process, response to external stimulus, response to cytokine, which are in agreement with the previous results reported by Zhao et al. (27). In addition, immune response and Type-1 interferon signaling were also the important enriched function in the endometrium tissue of pregnant bovine versus cyclic and nonpregnant (29). Similar results were also acquired by KEGG pathway analysis in this study, which revealed that immunity related pathways, such as herpes simplex infection, NOD-like receptor signaling pathway were the most notably enriched pathways. Meanwhile, some DEGs related with immunity and apoptotic process, such as *MR1*, *CASP8*, *OAS2*, *MX2*, *SP100*, were remarkably enriched in these pathways. All these experimental evidences suggest that immunity or apoptosis process induced by IFNT may play a significant role during early pregnancy in ruminants, which contributes to protect the embryo from rejection by maternal immune system.

It is well known that successful pregnancy in ruminants requires the participation of numerous genes regulated by IFNT. The results of predicted protein–protein interaction network demonstrated that a large number of DEGs of this study regulated directly or indirectly by IFNT and hormones constitute a complicated network, which might affect early pregnancy. As expected, the expression of many classical ISGs, such as *ISG15*, *RSAD2*, *MX2*, *OAS2* and *STAT1*, were upregulated by IFNT and hormones in this study. However, some DEGs have rarely been reported previously to be associated with pregnancy. Ubiquitin-specific protease 18 (USP18), a critical negative regulator of type I interferon signaling pathway by competing with JAK1 to bind interferon receptors, exert deubiquitination through the ISGylation modification system, and its expression is significantly enhanced in the endometria of pregnant cow on day 16 (30, 31). Similarly, a recent study proved that the expression of USP18 is significantly increased in goat uterine tissue from days 5–18 of pregnancy, which might promote the establishment of endometrial receptivity to affect early pregnancy by regulating the JAK/STAT1 and ISGylation pathway (32). Few studies have previously reported the relationship between proteasome subunit beta type-8 (PSMB8) and pregnancy. PSMB8 was found to be an important regulator in the process of glioma cell migration, proliferation and apoptosis mediated by ERK1/2 and PI3K-AKT pathways, which shares common pathways of invasion and angiogenesis with embryo implantation (33, 34). Xie et al. (35) proved that 3’-UTR of *PSMB8* targeted directly by chi-miR-451-5p of extracellular vesicles in goat uterine fluids might play an essential role in endometrial remodeling during peri-implantation. Apoptotic cell death has been shown to occur in the endometrium during mammalian preimplantation period, such as bovine (23, 36), rats (37), porcine (38). The expression of proapoptotic genes, *CASP8*, *XAF1* and *TNFSF10* were confirmed to be remarkably enhanced in the bovine endometrium during early pregnancy (36). These findings were also supported by a research conducted by Suzuki et al. (23), they demonstrated that apoptosis related genes (*CASP7*, *CASP8*, *FADD*) and pyroptosis related genes (*CASP4*, *CASP11*, *NLRP3*) are expressed at significantly higher levels in the endometrium of pregnant bovine at day 18 in comparison to non-pregnant. Meanwhile, the expression of *CASP7*, *FADD*, *CASP4*, *CASP11* and *GSDMD* were considerably increased in cultured uterine epithelial cells with IFNT administration (23).The similar results were observed in our study. However, the increased activity of caspases and elevation of apoptosis were barely detected in the endometrium of early pregnant bovine (23, 36), it is presumed that the proapoptotic signaling arose by IFNT are not transduced. Thus, the mechanisms protecting the endometrium from apoptosis to allow embryonic implantation might exist, that need to be further clarified.

Damage-regulated autophagy modulator 1 (DRAM1), an evolutionarily conserved protein located on the lysosome membrane, was identified as a direct target of p53 to modulate stress-induced autophagy and critical for p53-mediated apoptosis (39). Specifically, DRAM1 is involved in the process of autophagy under the conditions of various cellular stress, such as endoplasmic reticulum stress, mitochondrial damage and nucleic acid damage (40, 41), which was considered to be a key regulator related to the balance between cell survival and apoptotic cell death. Yang et al. (42) indicated that endoplasmic reticulum stress triggered by hormone and IFNT may contribute to early pregnancy success and the regulation of endometrial function by modulating mTOR-autophagy pathway in goat endometrial epithelial cells. Previous studies have suggested that DRAM1 might play an essential regulatory role during embryo implantation and early embryonic development. Chen et al. (43) confirmed the role of DRAM1 in mitophagy, which contributes to the regulation of preeclampsia caused by high levels of oxidative stress, mitochondrial dysfunction and apoptosis in the placental tissues of mice. *DRAM1* may be implicated in the regulation of proliferation, differentiation and apoptosis of placental cells, affecting the oxygen and nutrients supply of placenta. Nevertheless, the exact relationship between DRAM1 and early pregnancy, and the underlying mechanisms remain not well-understood. In this study, *DRAM1* was identified to be a key node gene interacting closely with other genes by constructing a PPI network based on STRING database, and confirmed to be a DEG by performing qRT-PCR verification. Therefore, we preliminarily explored the impact of *DRAM1* on expression of genes related with endometrial function. Prostaglandin-endoperoxide synthase 1 (*PTGS1*) and prostaglandin-endoperoxide synthase 2 (*PTGS2*) are rate-limiting enzymes in the biosynthesis pathway of PGs secreted by endometrium, which have crucial effects on the regulation of conceptus development and endometrial function (14, 44). Homeobox A10 (*HOXA10*) and Homeobox A11 (*HOXA11*) were proved to be involved in the regulation of endometrial receptivity, thereby increasing the embryo adhesion and implantation (45, 46). The results showed that mRNA expression of *HOXA11*, *PTGS1* and *PTGS2* were remarkably suppressed by silencing *DRAM1* under IFNT and hormone administration. Therefore, we presume that *DRAM1* may play a crucial role in early pregnancy by regulating endometrial function. Whereas, we did not further anatomize the specific molecular mechanisms by which *DRAM1* regulates endometrial function, that will be addressed in our future work.

## 5 Conclusion

Taken together, our results revealed multiple promising genes and signaling pathways in BEND, which might participate in the regulation of endometrial function during the early stage of pregnancy, especially early embryo implantation. The present study provides a more comprehensive overview of transcriptional changes of BEND in response to IFNT and hormones, which may pave the way for illustrating the molecular mechanisms of pregnancy recognition and endometrial function regulation, contributing to a better understanding of early pregnancy loss in ruminants.

## Data availability statement

The original contributions presented in the study are publicly available. This data can be found at: https://www.ncbi.nlm.nih.gov/; GSE253481.

## Ethics statement

The animal study was approved by the Institutional Animal Use Committee of Institute of Animal Science and Veterinary Medicine, Wuhan Academy of Agricultural Sciences. The study was conducted in accordance with the local legislation and institutional requirements.

## Author contributions

JY: Data curation, Formal analysis, Investigation, Methodology, Validation, Visualization, Writing – original draft, Writing – review & editing. CL: Data curation, Formal analysis, Investigation, Methodology, Writing – review & editing. HC: Formal analysis, Investigation, Methodology, Writing – review & editing. MX: Investigation, Methodology, Writing – review & editing. XH: Investigation, Methodology, Writing – review & editing. ZZ: Investigation, Methodology, Writing – review & editing. QL: Investigation, Methodology, Writing – review & editing. DW: Investigation, Methodology, Writing – review & editing. LC: Data curation, Formal analysis, Funding acquisition, Investigation, Methodology, Project administration, Writing – review & editing.
