# Supplementary figures and images for: Transcriptomic analysis of bovine endometrial epithelial cells in response to interferon tau and hormone stimulation
Source: Front Vet Sci. 2024 Feb 2;11:1344259. doi: 10.3389/fvets.2024.1344259 (PMC10873918; doi:10.3389/fvets.2024.1344259)

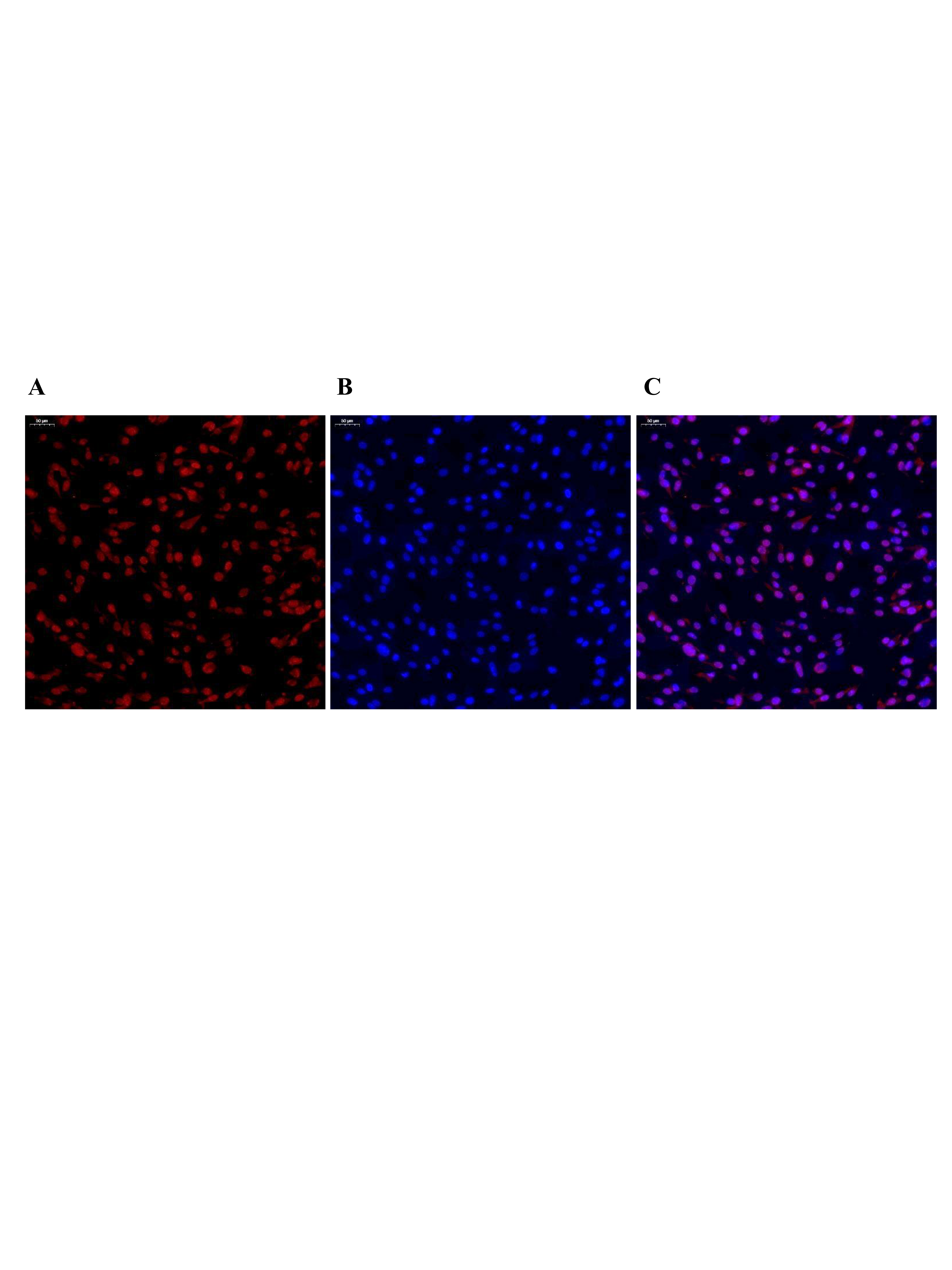

Supplement: Supplementary file 1 [file Image_1.TIF]

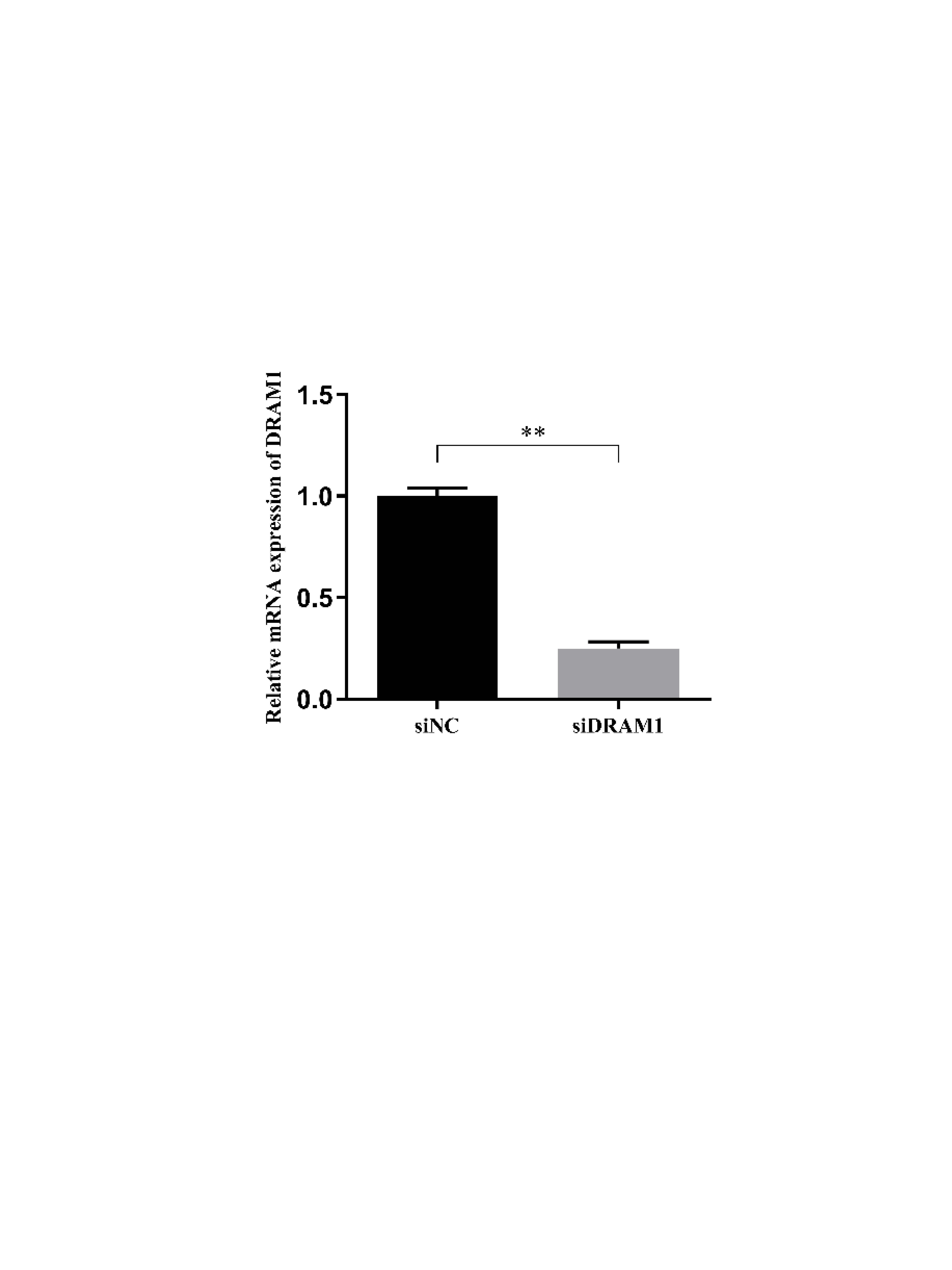

Supplement: Supplementary file 2 [file Image_2.TIF]
